# Effects of Landscape-Scale Environmental Variation on Greater Sage-Grouse Chick Survival

**DOI:** 10.1371/journal.pone.0065582

**Published:** 2013-06-18

**Authors:** Michael R. Guttery, David K. Dahlgren, Terry A. Messmer, John W. Connelly, Kerry P. Reese, Pat A. Terletzky, Nathan Burkepile, David N. Koons

**Affiliations:** 1 Department of Forest and Wildlife Ecology, University of Wisconsin, Madison, Wisconsin, United States of America; 2 Kansas Department of Wildlife and Parks, Hays, Kansas, United States of America; 3 Department of Wildland Resources, Utah State University, Logan, Utah, United States of America; 4 Idaho Department of Fish and Game, Blackfoot, Idaho, United States of America; 5 Department of Fish and Wildlife Sciences, University of Idaho, Moscow, Idaho, United States of America; 6 Northland Fish and Game, Whangarei, New Zealand; 7 Department of Wildland Resources and the Ecology Center, Utah State University, Logan, Utah, United States of America; University of Alberta, Canada

## Abstract

Effective long-term wildlife conservation planning for a species must be guided by information about population vital rates at multiple scales. Greater sage-grouse (*Centrocercus urophasianus*) populations declined substantially during the twentieth century, largely as a result of habitat loss and fragmentation. In addition to the importance of conserving large tracts of suitable habitat, successful conservation of this species will require detailed information about factors affecting vital rates at both the population and range-wide scales. Research has shown that sage-grouse population growth rates are particularly sensitive to hen and chick survival rates. While considerable information on hen survival exists, there is limited information about chick survival at the population level, and currently there are no published reports of factors affecting chick survival across large spatial and temporal scales. We analyzed greater sage-grouse chick survival rates from 2 geographically distinct populations across 9 years. The effects of 3 groups of related landscape-scale covariates (climate, drought, and phenology of vegetation greenness) were evaluated. Models with phenological change in greenness (NDVI) performed poorly, possibly due to highly variable production of forbs and grasses being masked by sagebrush canopy. The top drought model resulted in substantial improvement in model fit relative to the base model and indicated that chick survival was negatively associated with winter drought. Our overall top model included effects of chick age, hen age, minimum temperature in May, and precipitation in July. Our results provide important insights into the possible effects of climate variability on sage-grouse chick survival.

## Introduction

Selective pressures result in the evolution of a life history conducive to species persistence under the environmental conditions encountered throughout the species' evolutionary history. Environmental conditions are not static, but rather experience climatic, geological, and successional changes through time. While such changes continue to occur naturally, anthropogenic disturbances have critically altered many of these processes, resulting in environments changing at rates that exceed the ability of some species to adapt [1]. The impact of rapidly changing environments may be particularly severe for species with limited dispersal opportunities (i.e., those existing in highly fragmented habitats; [2]). Efforts to conserve such species must focus on identifying the key demographic rates that are limiting population growth and the environmental factors that affect these rates [3].

During the 20th century, greater sage-grouse (*Centrocercus urophasianus*; hereafter sage-grouse) populations experienced precipitous declines as a result of anthropogenic habitat destruction, degradation, conversion, and fragmentation [4], [5]. In response to declining populations and increasing threats to remaining habitat, the Canadian Committee on the Status of Endangered Wildlife in Canada declared sage-grouse to be an endangered species in 1998 [6]. The United States Fish and Wildlife Service (USFWS) designated the sage-grouse as a candidate for protection under the Endangered Species Act in 2010 [7].

Sage-grouse are endemic to sagebrush (*Artemisia* sp.) dominated habitats of western North America, which have historically been very stable given that sagebrush is a long-lived and persistent plant. As such, sage-grouse evolved to use sagebrush for food and cover throughout the majority of their annual cycle. However, sage-grouse chicks do not consume sagebrush during their early development but instead require forbs and their associated arthropod communities. These components of the sagebrush ecosystem are highly dependent upon precipitation levels and therefore may exhibit high interannual variability. Thus, sage-grouse evolved a life history characterized by high annual adult survival but relatively low and variable reproductive rates compared to most other tetronids [8], [9].

Recently, researchers have applied life cycle models to gain a better understanding of factors affecting greater sage-grouse at the population [10] and range-wide scales [9]. Although both studies found sage-grouse population growth rates to be most sensitive to variability in adult female survival, they also found chick survival to have the second largest impact on population growth. While numerous studies have evaluated factors which influence survival rates of adult female sage-grouse [11], [12], little is known about factors affecting chick survival. Generally, demographic rates to which the population growth rate is highly sensitive have low temporal variability [13], [14]. Thus, chick survival should exhibit greater inter-annual variability and could therefore contribute more to spatio-temporal variability in population growth rate [15] even though sage-grouse populations are more sensitive to hen survival.

Previously published studies of factors affecting sage-grouse chick survival [16], [17], [18] have focused on micro-scale habitat factors such as percent coverage and height of forbs and grasses and availability of arthropods at chick location sites. These studies follow logically from previous research on sage-grouse brood habitat selection [19], [20], [21], [22] and chick diets [23], [24], [25], [26]. Collectively, these studies clearly demonstrate that broods typically select relatively mesic habitats with abundant forbs and arthropods and that these choices are related to chick survival. However, existing studies have not investigated the impacts of large-scale environmental processes (drought, temperature, etc.) on sage-grouse chick survival.

Landscape-scale environmental factors such as habitat condition, drought, and climate may be correlated with chick survival. Normalized Difference Vegetation Index (NDVI) is a commonly used index of plant production and habitat quality [27], [28], [29], [30], with higher values of the index corresponding to increased levels of “greenness”. Despite being less sensitive to plant phenology in sagebrush steppe ecosystems [29] and potential biases due to image quality, NDVI has been shown to be positively related to sage-grouse recruitment and population growth [27]. Drought and climatic variables can work independently and in concert to affect habitat parameters and can be reflected in NDVI values. For example, measures of drought, precipitation, and temperature can be correlated to winter snow pack which is known to be a major driver of vegetation dynamics throughout much of the mountainous regions of western North America [31]. However, climatic variables may affect sage-grouse chick survival in ways other than through their influence on habitat quality. Young grouse may be susceptible to exposure mortality during periods of extreme temperatures [32]. Additionally, numerous studies have documented increased nest and chick predation rates following precipitation events (i.e., moisture facilitated predation hypothesis; [33], [34], [35]). This effect is typically attributed to increased scent production resulting from increased bacterial growth when skin and feathers are wet [36]. Although the assumptions underlying the moisture facilitated predation hypothesis have not been thoroughly evaluated in the context of the hypothesis, the processes of moisture facilitating microbial activity and increased microbial activity resulting in increased scent production have been well documented in other fields of study [37], [38], [39], [40].

The objective of our study was to model the effects of landscape scale biotic (habitat greenness) and abiotic (climate and drought) factors on sage-grouse chick survival. We demonstrate the utility of data that can be readily obtained for virtually any geographic region or temporal period via web-based resources for predicting sage-grouse chick survival.

## Materials and Methods

### Study areas

**Figure 1:**
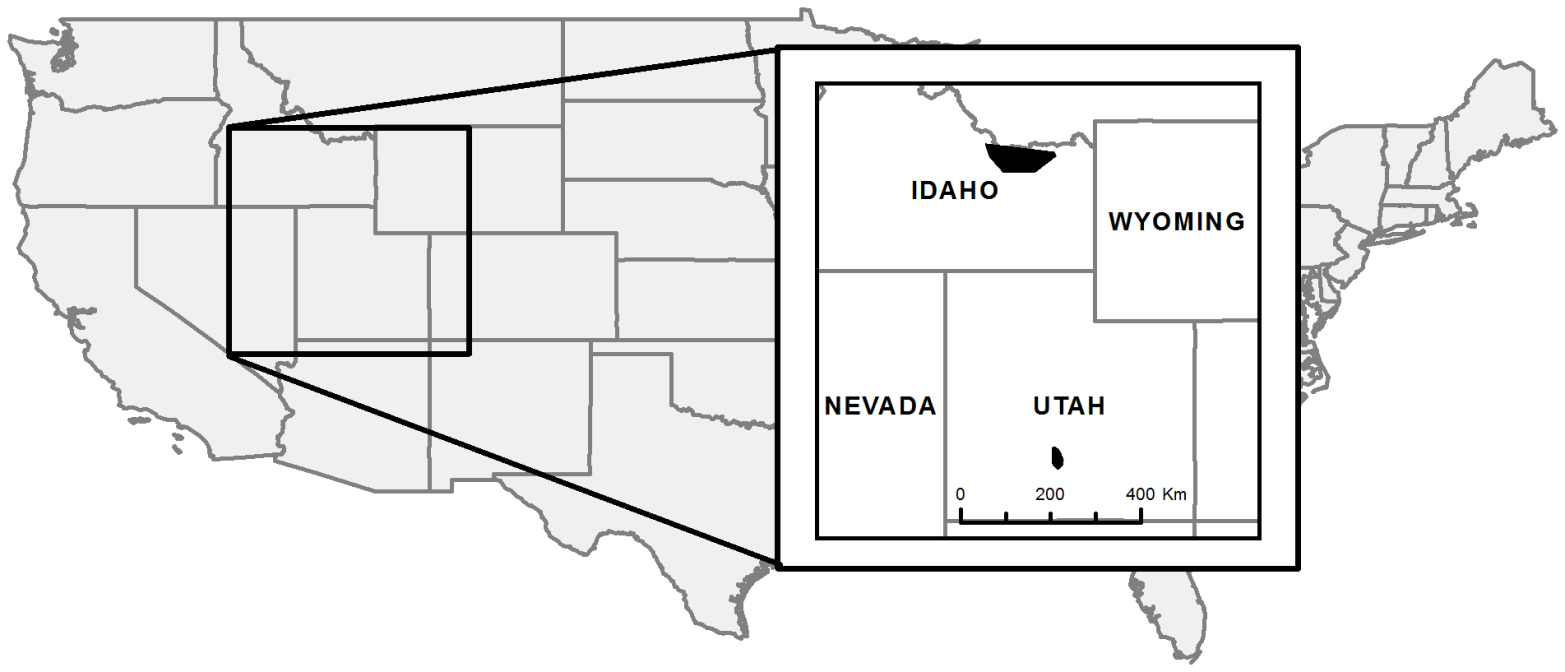
Map of study areas in Idaho and Utah.

Data were collected as part of 2 larger studies conducted in Idaho and Utah (Fig. 1). The Idaho study was conducted from 1999–2002 in sagebrush-grassland habitats of the Upper Snake River Plain in southeastern Idaho (44°13′N, 112°38′W). This area was characterized by relatively low topographic relief with elevation across the site ranging from 1300–2500 m. Approximately 50% of the area was privately owned, with the remainder being public lands administered by the U.S. Bureau of Land Management (BLM). Annual precipitation varied by elevation with low elevation areas receiving 17.5 to 30.0 cm of precipitation. Most sage-grouse habitat at lower elevations was dominated by Wyoming big sagebrush (*A. tridentata wyomingensis*). At higher elevations precipitation ranged from 30.5 to 45.5 cm annually and the habitat was dominated by mountain big sagebrush (*A. t. vaseyena*). Livestock grazing and cropland agriculture were the dominant land uses across the area [41].

The Utah study area was located on Parker Mountain in south-central Utah (38°17′N, 111°51′W). Research on sage-grouse chick survival was conducted on this site from 2005 through 2009. The area encompassed 107,478 ha and was administered by the Utah School and Institutional Trust Lands Administration (40.8%), United States Forest Service (20.2%), BLM (33.9%), and private ownership (5.1%). Parker Mountain is a sagebrush-dominated plateau at the southern edge of the sage-grouse range. It is one of the few areas remaining in Utah with relatively stable numbers of sage-grouse, and it includes some of the largest contiguous tracts of sagebrush in the state [42]. Grazing by domestic livestock is the predominant land use practice across the study site. The area receives between 40 and 51 cm of precipitation annually, which generally exhibits a bi-modal pattern, occurring either as rain during the seasonal monsoonal period from late summer and early autumn or as snow during winter.

### Field methods

We captured female sage-grouse on and around leks using spotlights, binoculars and long handled nets [43], [44] during early spring (March–April). Captured hens were classified as being either second-year (SY) or after second-year (ASY) birds based on wing characteristics as described by Beck et al. [45]. Birds were fitted with 15–19 g necklace style radio-transmitters (Advanced Telemetry Systems, Isanti, MN, USA; Holohil Systems, Carp, Ontario, Canada) and released at the capture location.

Marked hens were monitored during April and May to determine if they initiated a nest. Nesting was confirmed visually, but hens were never intentionally flushed from their nest due to the tendency of female sage-grouse to abandon their nest if disturbed [46], [47]. Nesting hens were visually monitored every 2–3 days to determine nest fate. Nests were monitored daily as the anticipated hatch date approached.

We captured chicks by using telemetry equipment to locate radio-marked hens. During capture events, the brood hen was flushed and chicks were captured by hand and placed in an insulated container to help maintain body temperature. We captured most broods within 48 hours of hatching with all broods being captured within 1 week of hatching. Captured chicks were weighed to the nearest gram and marked with a ≤1.5 g backpack-style radio-transmitter (Advanced Telemetry Systems, Insanti, MN in 1999–2001 and 2005, Holohil Systems, Carp, Ontario, Canada in 2006–2008, and American Wildlife Enterprises, Monticello, FL in 2009) attached with 2 sutures [48]. For the Idaho study site, 2–3 chicks per brood were selected at random to receive radio-transmitters. At the Utah study area, we marked all captured chicks except in 2006 when 3 chicks from each brood were randomly selected to receive transmitters. Chicks found dead in the immediate vicinity of the capture site were considered to have died as a result of handling and were excluded from subsequent analyses. Broods were typically checked within 12 hours of being marked and all chicks classified as capture mortalities were found intact within a few meters of the release site, indicating that their death was directly attributable to the capture event. Our decision to exclude chicks classified as capture mortalities from our analysis may have inflated survival estimates if some of these mortalities were in fact not related to capture. However, we do not believe this was a common occurrence if it occurred at all.

Marked chicks were located every 1–2 days until they reached 42 days of age. Monitoring intervals did occasionally exceed 2 days due to inclement weather events or difficulties locating broods following large movements. Extensive efforts were made to find any chicks missing from a brood. We occasionally recovered chick transmitters with no chick remains or signs of predation. These recoveries were classified as mortalities although it is possible that transmitters may have been lost for reasons other than chick death. Alternatively, we could have right-censored these specific events. While this would have been a valid option, we chose to treat the events as mortalities to ensure that our survival estimates were conservative. Due to the difficulty of distinguishing predation from scavenging, we did not assign specific causes of mortality.

All necessary permits were obtained for the described field studies. Permission to capture and mark sage-grouse in Idaho was obtained from the Idaho Department of Fish and Game and from the Utah Division of Wildlife Resources for the Utah study site. Grouse capture and transmitter attachment procedures were approved under the Utah State University Institutional Animal Care and Use Committee (IACUC) permit #945R and #942 and University of Idaho IACUC permit #2000-7.

### Covariate data

We compiled year and site specific covariate data pertaining to drought, landscape greenness, and climate. We included seasonal (preceding winter and current summer) and monthly (May–July) Palmer Drought Severity Index (PDSI) and Palmer Z-Index (PZI) values. For climate and drought covariates, we defined winter as the period from 1 November to 30 April because precipitation would likely fall as snow on both study sites during these months. Summer was defined as the period from 1 May through 31 July. We did not include August because very few broods were monitored beyond July.

While both the PDSI and PZI indices are measures of drought and their values interpreted similarly (negative values correspond to drought conditions while positive values indicate wet conditions), the PDSI is most appropriate for measuring conditions across long time periods (several months) while the PZI is designed to measure conditions across shorter time periods (several weeks to a few months, [49]). Although drought is often thought about in terms of the presence or absence of precipitation, PDSI and PZI also account for site specific rates of evapotranspiration, soil moisture recharge, runoff, and moisture loss [50]. Additionally, both drought indices are calculated relative to the long-term average drought conditions at a specific site. As such, values of each index are standardized to have a common interpretation across locations [50]. All drought data were downloaded from the National Oceanic and Atmospheric Administration's National Climate Data Center (http://www.ncdc.noaa.gov/temp-and-precip/time-series/index.php).

Climate variables of interest included total precipitation, minimum temperature, and maximum temperature for the same seasonal and monthly periods described above. Unlike drought covariates, climate covariates were not adjusted to account for other physical processes or long-term site-specific averages. Because complete and representative weather station data were not available for both study sites, we used the Parameter-elevation Regressions on Independent Slopes Model (PRISM; http://www.prism.oregonstate.edu/) to estimate climatic data for both sites. PRISM is a knowledge-based climate analysis system capable of generating gridded predictions of climate data from known point climate data and a digital elevation map [51]. We used ArcMap10 to generate minimum convex polygons around all chick locations at both sites to define our study sites. We then extracted climate variable data from the corresponding PRISM layer.

Phenological change in landscape greenness was measured using NDVI for each study area. We generated NDVI values using Landsat 4–5 satellite images obtained from the United States Geological Survey EarthExplorer website (http://earthexplorer.usgs.gov/). We selected images captured between 1 May and 31 August with minimal cloud coverage. Due to variability in image quality (i.e., cloud cover) and capture date, we were not able to use images taken on identical dates across years. Images were processed using the ERDAS Imagine remote sensing image analysis software (Intergraph, Madison, AL, USA) to apply radiometric corrections that eliminated background noise while retaining temporal variance in vegetative reflectance [52]. We used ERDAS Imagine to calculate NDVI on a pixel-by-pixel basis for each image based on the ratio of red to near-infared reflectance [53]. For each site, we fit our observed NDVI values to a linear model:
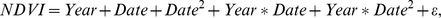
to estimate daily NDVI values where 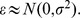 This model provided a good fit to the data (*R* ^2^>0.80, *F*_11_>4.50, *P*<0.001). We used predicted values to estimate mean and maximum NDVI values for May, June, July, and summer (as defined above). We also estimated the mean NDVI value at the date of hatching, 15 days before hatch, date of each survival observation, and 5 and 10 days prior to the date of each observation. Finally, because variability in NDVI was low due to sagebrush obscuring the phenological progression of forbs and grasses, we adjusted all NDVI values by subtracting out the year and site specific NDVI value on 1 May. This linear transformation effectively removed baseline site and year variation, thereby allowing our analysis to focus more directly on the effect of within-year plant phenology at a site.

### Analysis

Missing chicks whose fate could not be determined were removed (i.e., right-censored) from the data set at the time of their last confirmed detection. Failure to locate chicks may have been the result of transmitter failure, the chick being removed from the study site by a predator, or long distance movements that exceeded the range of the transmitters. On a few rare occasions, chicks were found alive several weeks after going missing. The flexibility of our model allowed us to reintroduce these chicks back into the data set once rediscovered. Alternatively, we could have assumed that missing chicks were either dead or alive but our approach likely provides the most realistic estimate of chick survival because only chicks with known fate were allowed to influence daily survival rates [17].

We modeled sage-grouse chick daily survival rates from hatch to 42-days of age using the known-fate maximum likelihood estimator developed by Manly and Schmutz [54] and extended by Fondell et al. [55]. This model assumes a piecewise survival function such that the survival rate from age *t* to age *t*+1 is:
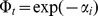
where *α_i_*≥0, for *t*_i-1_≤*t*<*t*_i_, with t_0_ = 0, and i = 1,2,…,p. Therefore the daily survival rate (DSR) for ages 0 to *t*_1_ days is assumed to be exp(-α_1_), the DSR for ages *t*_1_ to *t*_2_ days is assumed to be exp(-α_2_), and so forth, with p survival intervals. If N_a_ chicks are observed in a brood at age *a* then the number of survivors at age *b*>*a*, N_b_, has a binomial distribution with mean:
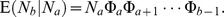
To account for extra-binomial variance, the variance term is:
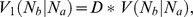
Where D is a constant and V(N_b_|N_a_) is the binomal variance given by:
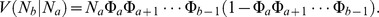
This variance formulation assumes that most extra-binomial variation is the result of lack of independence in the fates of chicks within broods. Given this formulation and assumptions, the log-likelihood function for the observed number n_b_ at the end of a survival period is derived from the normal density function and takes the form:
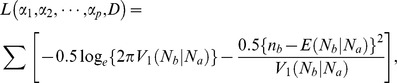
where the summation is over all the instances in the data set where a brood size is observed at time *a* and then observed next at time *b* [54].

This generalized linear model is appropriate because it allows for variable observation intervals, changes in brood size due to missing chicks, and accounts for lack of independence in fates among chicks within a brood by using a quasi-likelihood approach [17], [54], [55]. Values of D near 1 indicate minimal dependence in the fates of brood mates whereas larger values correspond to decreasing independence among brood mates [54]. Covariates were modeled using a logit-link. Maximum likelihood estimates for all parameters were estimated using the ‘OPTIM’ function in R 2.14.1 [56].

To examine processes affecting chick survival in our populations, we first developed models that included alternative parameterizations of chick age. For example, we created models with categorical age classes wherein the categories were based on biological development of chicks, such as pre- versus post-flight ages or early ages when the diet consists primarily of insects versus later ages when forbs become important. We also considered linear and quadratic models of age treated as a continuous variable. Competing models of the various chick age parameterizations were ranked using the quasi-likelihood version of Akaike's Information Criterion adjusted for sample size (QAIC_c_: [57], [58]). Models with ΔQAIC_c_≤2 were considered to be equally supported by the data, and when this occurred we applied the principle of parsimony and based our inference on the model with the fewest parameters [59]. Upon identifying the best parameterization of chick age, we next considered the addition of hen age (SY or ASY) and hatch date effects, as both have been shown to be important predictors of sage-grouse chick survival [17], [60]. Year and site effects were not modeled explicitly because all covariates of interest were site and year specific (i.e., site and year effects were modeled implicitly). The validity of the approach was assessed by adding year and site effects to our final top model and monitoring the change in QAIC_c_.

We then developed candidate model sets for each of the 3 covariate groups. Covariates within each group tended to be correlated. To insure the interpretability of parameter estimates (i.e., to avoid multicollinearity), covariates with a Pearson correlation coefficient (ρ) greater than 0.50 were not included in the same model. To determine which of the 3 groups of covariates had the greatest impact on chick survival, we did not include covariates from different groups in the same model. These restrictions limited the complexity of models we considered. Upon identifying the top model for each of the 3 covariate groups, we obtained 95% bootstrap confidence intervals for model parameters using 5,000 samples with replacement from our dataset [61]. All continuous covariates were Z-standardized prior to analyses. We calculated the proportional reduction in deviance [62] for each model relative to the null model:
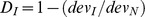
where *D_I_* is the Zheng-score for the model of interest, *dev_I_* is the deviance for the model of interest, and *dev_N_* is the deviance for the null model (unless otherwise noted, an intercept-only model) and deviance was calculated as -2*quasi-log-likelihood. The Zheng-score is a goodness-of-fit measure for generalized linear models of longitudinal data and can be interpreted similarly to a standard coefficient of determination, R^2^, in a linear model [62]. We then further assessed model fit by calculating the ratio of the Zheng-score for the model of interest relative to the spatially and temporally saturated model [63]:
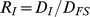
where *D_I_* is the Zheng-score for the model of interest and *D_FS_* is the Zheng-score for the fully spatial and temporally saturated model. Values of R close to zero indicate little improvement in model fit over the null model, whereas values of R that approach one indicate model fit similar to the fully saturated model.

## Results

### Chick statistics

Most hens had a single brood during the course of our multi-year study; however, 24 of the 142 hens had broods during more than one year of the study. Peak hatch date ranged from 25 May to 7 June at the Utah study area and from 19 May to 30 May for the Idaho area. During the 9 years of study we attached radio transmitters to 518 chicks from 142 broods, resulting in 11,188 chick exposure days (Table 1). Chick age at the time of capture ranged from 1 to 8 days. A total of 18 chicks were determined to have died as a result of capture, and were excluded from analyses. We censored an additional 159 missing chicks from the dataset after the last date of telemetry observation.

**Table 1:** Capture statistics for greater sage-grouse chicks marked in Idaho (1999–2002) and Utah (2005–2009).

Year	Broods^1^	Chicks^2^	Hen Ages^3^	Marked^4^
1999	13	30	SY = 3, ASY = 10	2.31
2000	15	42	SY = 4, ASY = 11	2.80
2001	14	40	SY = 1, ASY = 13	2.86
2002	24	71	SY = 5, ASY = 19	2.96
2005	21	89	SY = 11, ASY = 10	4.24
2006	21	61	SY = 0, ASY = 21	2.90
2007	12	55	SY = 4, ASY = 8	4.58
2008	11	66	SY = 2, ASY = 9	6.00
2009	11	64	SY = 1, ASY = 10	5.82
Total	142	518	SY = 31, ASY = 111	3.65

1 Number of broods captured.

2 Total number of chicks marked with radio-transmitters.

3 SY = second year hen (hatched the previous year), ASY = after second year hen (hatched ≥2 years earlier).

4 Average number of chicks marked per brood.

### Base null model

Our best intra-annual model of chick survival included linear and quadratic effects of chick age (Table S1). This model clearly out-performed all other intra-annual temporal models in terms of QAICc value. This model was then used as the base model for evaluation of the main effects of hen age and hatch date. Comparison of QAICc scores for these models (Table S2) shows that the model including only hen age and the additive effects of hen age and hatch date were both competitive (Δ QAICc<2). Because the model containing only the effect of hen age was more parsimonious, we chose to retain this model as the base null model for comparison of climate, drought, and greenness phenology covariates [64].

### NDVI models

All NDVI covariates considered were highly correlated (all ρ>0.62). Thus, we did not construct models containing multiple NDVI covariates. All 13 single NDVI-effect models produced positive beta estimates (Table S3). Five models were equally supported by the data (ΔQAICc<2.0, Table S3). However, none of the 13 models, including the top 5 models, resulted in a meaningful increase in model fit (as measured by the R-score) relative to the base model. Additionally, the 95% confidence intervals for the effect of the average NDVI in July relative to May 1 for a given site and year (the model with lowest QAIC_c_) were symmetrical around zero, indicating a weak and imprecise effect (Table S4).

### Drought models

**Figure 2:**
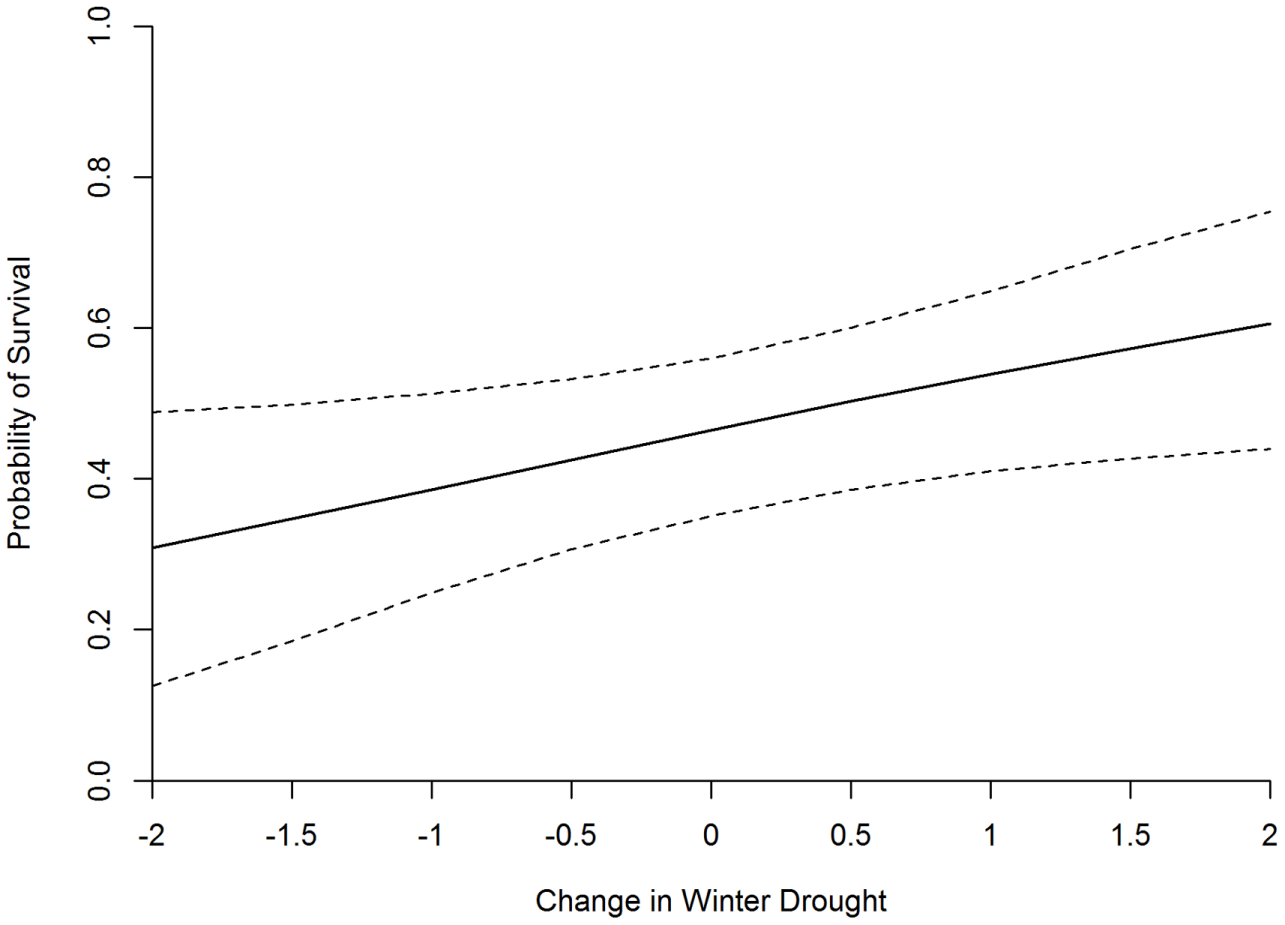
Effects of changes in winter drought severity (PZI) on the probability of greater sage-grouse chick survival to 42 days of age. Dashed lines indicate 95% confidence intervals. Negative values correspond to increasingly severe drought conditions. A change of 0.0 is equal to the mean Winter Palmer Z-Index score observed during the extent of this study. Palmer (1965) stated that a drought score of -2 was indicative of moderate drought.

As with our NDVI covariates, all drought covariates were highly correlated (all ρ>0.77) so only single-effect models were considered. Of the 10 drought models considered, the model including the effect of the PZI for the preceding winter performed best (Table S5). The addition of winter PZI to the base model resulted in an approximate 40% increase in the R-score, indicating a substantial improvement in model fit. Further, the 95% confidence interval for winter PZI indicated a significant positive effect of the covariate on chick survival (Fig. 2, Table S6).

### Climate models

**Figure 3:**
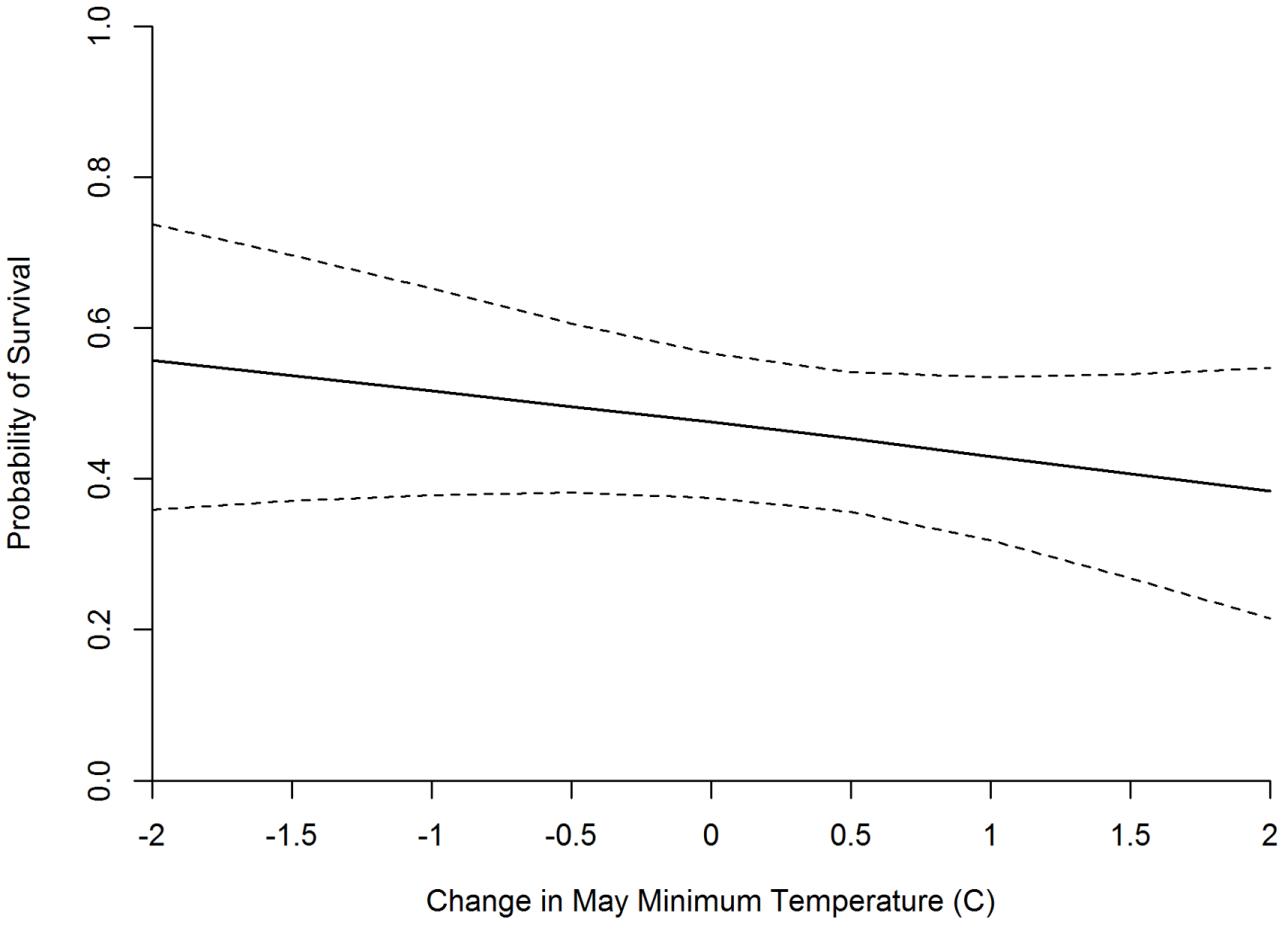
Effects of May minimum temperature on the probability of greater sage-grouse chick survival to 42 days of age. Dashed lines indicate 95% confidence intervals. A change of 0.0 is equal to the mean May minimum temperature observed during the extent of this study.

**Figure 4:**
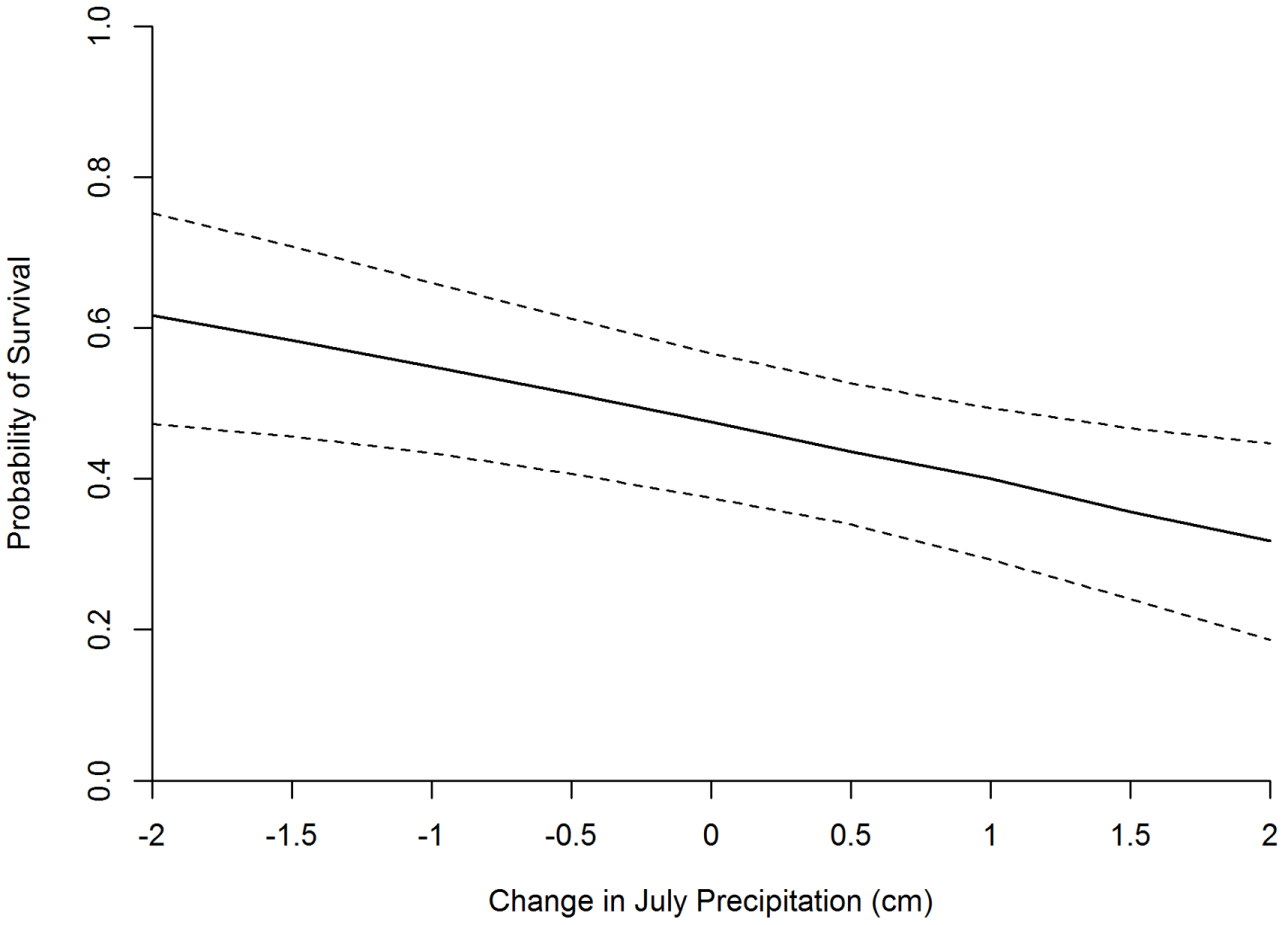
Effects of July precipitation on the probability of greater sage-grouse chick survival to 42 days of age. Dashed lines indicate 95% confidence intervals. A change of 0.0 is equal to the mean July precipitation observed during the extent of this study.

Several combinations of climatic covariates had correlation coefficients below our critical value. Additive effects of multiple climatic covariates were modeled if the correlation between all covariates was less than 0.5 and the model was deemed to be ecologically meaningful. These conditions resulted in the construction of 18 models (Table S7). The top climate model (minimum temperature in May+total precipitation in July) fit the data well (R-score = 0.766) and was the overall top model (Table 2). Both climatic effects in the top model were negatively associated with chick survival (Figs. 3–4). Despite the model fitting the data well, only the effect of July precipitation was significantly different from zero (Table S8). To ensure that the effects in our top model were robust and were not confounded by underlying effects of site and/or year, we added site and year effects to our top climate model (Table S9). Models containing year effects did not converge and models including additive and interactive site effects were not supported by the data (based on ΔQAIC_c_), indicating that our results are robust across the 2 study sites. Allowing daily survival to change with chick age and holding all other covariate values at the sample mean, predicted values from our top model yielded a 42-day survival probability of 0.475 (95% CI = 0.375 to 0.566). Estimates of *D* from the 3 top models all indicate that dependence in the fates among brood mates was low (1.6149 to 1.7085) but non-negligible (Tables S4, S6, S8).

**Table 2:** Comparison of top chick survival models among the landscape-scale covariate groups.

Model	K	QAICc	ΔQAICc	w_i_	R-score
Base+Saturated Model	13	−121.45	0.00	0.999	1.000
Base+May Min Temp+July Precip (−,−)	7	−58.30	63.15	0.000	0.766
Base+Winter PZI (+)	6	58.21	179.66	0.000	0.396
Base+July Mean NDVI (+)	6	178.53	299.98	0.000	0.022
Quadratic Chick Age+Hen Age (Base)	5	183.48	304.93	0.000	0.000

All models contain the base effects of quadratic chick age and hen age. Models were evaluated using the Quasi-Akaike's Information Criterion (QAIC). K = number of parameters. w_i_ = model weight (i.e. the likelihood of a particular model being the best model). R-score = percent reduction of deviance relative to the base model (Quadratic Chick Age+Hen Age). The saturated model contains effects for site (1 parameter) and each year (7 parameters). Typically 8 parameters would be required to model the effects of 9 years. However, because years did not overlap between the 2 sites we were able to fully specify year effects with only 7 parameters.

## Discussion

Studies of avian survival are often short-term and conducted on a single study area. While such studies provide important information, for many species there is a lack of knowledge concerning general large-scale factors which influence dynamics across space and time. An understanding of large-scale population drivers is essential for effective wildlife conservation planning and provides a baseline for developing meaningful hypotheses about specific local factors affecting populations at smaller spatial and temporal scales. Our study is the first to attempt to establish this baseline for the survival of greater sage-grouse chicks across multiple populations.

### Independence of brood mates

Our modeling approach allowed simultaneous incorporation of commonly collected demographic information (hatch date, chick age, hen age) as well as publically accessible landscape level biotic (NDVI) and abiotic (temperature, precipitation, drought) information into survival models implemented in R [56]. Additionally, our approach allowed us to account for the potential lack of independence among chicks from the same brood [55]. Estimates of *D* from the top 3 models (Tables S4, S6, S8) ranged from 1.6149 to 1.7085 and, in all cases, confidence intervals did not include one or the mean number of chicks marked per brood (3.65, Table 1). This finding indicates that, while not highly dependent, fates were not independent among brood mates. This supports our decision to use the Manly and Schmutz [54] survival estimator rather than traditional known-fate survival estimators that assume fates of individuals are independent.

### Survival rate

Overall, chick survival during this study was relatively high. Our top model produced an average 42-day survival probability of 0.475 (95% CI = 0.375 to 0.566). This is similar to the 42-day survival rate of 0.50 reported for sage-grouse chicks by Dahlgren et al. [17] and considerably higher than the 28-day survival rate of 0.392 reported by Gregg and Crawford [18]. However, comparison of our observed survival rate to those of Gregg and Crawford [18] are potentially confounded by the use of different transmitter attachment methods (suture attachment versus subcutaneous implant). Gregg and Crawford [18] report a total of 32 chick mortalities attributable to capture compared to only 18 in our study despite a similar total number of chicks being marked in both studies. It is possible that our survival rates may be inflated if some chicks treated as capture mortalities were incorrectly classified as such. However, the low incidence of chicks being classified as capture mortalities makes it unlikely that any misclassifications would significantly influence our findings.

Our analysis supported previous research that has shown both chick age and hen age to be important predictors of sage-grouse chick survival [17], [60] (Tables S1 and S2). Interestingly, our models indicate that chicks hatched to second-year hens experience higher survival rates than chicks hatched to older hens. This effect has been previously reported for sage-grouse [17], [60] although the mechanism underlying it has not been thoroughly explained. Despite being poorly estimated throughout (Tables S4, S6, S8), we chose to retain chick and hen age covariates in all models to minimize bias in estimates of the effects of interest.

### Effects of NDVI

In recent years, NDVI has proven to be a useful tool for understanding various aspects of animal ecology [65]. We found positive relationships between all of our measures of NDVI phenology and chick survival (Table S3). However, none of the NDVI measures resulted in substantial improvements in model fit, as measured by the R-score, relative to the base (chick age+hen age) model, nor were the effects significant. Blomberg et al. [27] similarly found that NDVI was positively associated with sage-grouse recruitment and population growth, but that NDVI provided weak predictive power relative to other predictors such as precipitation.

Given the importance of invertebrates and herbaceous vegetation in the diet of sage-grouse chicks [23], [18], [25], the poor predictive power of NDVI for sage-grouse chick survival is somewhat surprising because NDVI is a well-established index of net primary production [65], [66], [67], and invertebrate production is positively related to plant production [68]. We suggest that the extensive coverage of sagebrush across both study sites resulted in phenological measures of NDVI being less sensitive to changes in coverage of forbs and grasses, thereby diminishing the ability of NDVI to measure changes of direct relevance to sage-grouse chicks. Correspondingly, Paruelo and Lauenroth [29] found a generally smaller range of NDVI values in sagebrush-steppe ecosystems than in grasslands where plant phenological changes are likely easier to detect.

### Effects of drought

Although local availability and abundance of specific invertebrates and forbs is proximally related to sage-grouse chick survival [17], [18], survival is likely under the primary influence of physical factors such as precipitation (amount and timing), temperature, and drought. Accordingly, our analysis indicated that abiotic factors were better predictors of sage-grouse chick survival than phenology of NDVI. Our top drought model (Table S5) indicated the presence of a significant relationship between winter drought and chick survival (Table S6). Since smaller PZI (and PDSI) values correspond to increasingly severe drought conditions, the positive parameter estimate associated with the winter drought effect implies that winter droughts lead to reduced chick survival (Fig. 2). Unfortunately, our data do not allow us to identify the true causal mechanism(s) underlying this relationship. Schwinning et al. [69] found that winter drought, even more so than summer drought, affects plant production during the following summer. Therefore, winter drought may affect sage-grouse chick survival via its influence on brood habitat quality. Additionally, winter drought may influence chick survival by affecting resource provisioning during egg formation. Forb abundance during the pre-nesting period is positively associated with hen nutrition [70], and hen nutrition prior to nest initiation is positively related to reproductive investment [71]. Thus, we suggest that either or both of these effects may be the mechanism behind the relationship between winter drought and chick survival that we observed.

### Effects of climate

Blomberg et al. [27] reported relatively stable survival rates for adult sage-grouse but found that recruitment was variable and strongly influenced by annual climatic variation. These findings led the authors to conclude that stability of sage-grouse populations is dependent upon stable annual survival rates and occasional large inputs of new individuals into the population when climatic conditions are amenable to chick and juvenile survival. Our results support this assertion that climatic variables play a primary role in determining sage-grouse reproductive success. Of the 3 groups of predictors of chick survival we considered, models containing climatic effects clearly outperformed all other models (Table S7 versus Tables S5 and S3).

Our top climatic model fit the data well (Table 2, R-score = 0.766). In addition to the effects of chick and hen age, the top model included the minimum temperature in May (MMT) and precipitation in July, both producing negative parameter estimates (Table S8). We initially hypothesized that MMT could have either a positive or negative association with chick survival. Specifically, we predicted that MMT could be positive if higher minimum temperature resulted in fewer chicks dying due to exposure. Alternatively, we predicted that a negative effect of MMT would be attributable to high minimum temperatures leading to early snow melt and thus lower soil moisture and poor habitat quality throughout the brood-rearing period. We conclude that the latter is the case. Although particularly cold temperatures in late May could potentially result in increased exposure mortality, consideration of our peak hatch dates (see Section 3.1) reveals that it is unlikely that many chicks would be hatched early enough to be exposed to extreme low temperatures likely occurring in the first half of May. We also note that the minimum temperature in June is positively associated with chick survival, possibly indicating that exposure mortality does increase as temperature decreases during this timeframe.

Our interpretation of the negative effect of MMT on chick survival does raise concerns about the impact of projected climate on future sage-grouse reproductive success. Significant temperature increases have been documented across western North America in recent decades, and climate models consistently predict that temperatures will continue to increase into the foreseeable future [72]. Observed and projected warming trends have also been connected to observed and projected transitions from winter precipitation falling as rain rather than snow and consequently reduced spring snow pack [72], [73], [74].

The trend in warming temperatures could impact sage-grouse population dynamics as a result of phenological asynchrony [75], increased spread of exotic species such as red fox (*Vulpes vulpes*; [76]) and cheatgrass (*Bromus tectorum*; [5]), and increased frequency and severity of wildfires [77]. Blomberg et al. [27] concluded that projected climate change could result in reduced recruitment of sage-grouse. Our results support this conclusion. Figure 3 shows model-derived chick survival estimates across a range of MMT. According to our results, a 2°C increase in mean MMT, well within the range projected by most climate models [72], will result in an approximate 10% reduction in sage-grouse chick survival. This effect could be mitigated if sage-grouse are able to adjust their hatch dates to correspond with earlier snow melt and advanced plant phenology. A simple linear regression of our observed median hatch dates on MMT shows a significant correlation (p = 0.0171, R^2^ = 0.58). This demonstrates that sage-grouse may be capable of synchronizing the timing of nesting with MMT for at least the range of MMT observed during our study. However, it is not clear if the level of plasticity in breeding phenology is sufficient to compensate for future climatic changes. Additionally, if warming results in a shift in the form of winter precipitation from snow to rain [73], chick survival may still be negatively affected by poor habitat quality, even if hens are able to adjust nest initiation to correspond with early snowmelt.

We initially hypothesized that July precipitation (JP) would have a positive effect on sage-grouse chick survival due to a moisture associated increase in plant and arthropod forage. However, our analysis showed a significant negative effect of JP (Table S8). While this result may be less intuitive, we conclude that it is real and meaningful. At least 2 mechanisms may underlie the relationship between chick survival and JP. First, chicks may be susceptible to exposure mortality in July. As noted above, the Utah study area is located in a monsoonal zone and receives a substantial proportion of the annual precipitation during late summer (primarily July and August). Monsoonal storms across the Utah study area often build quickly and result in significant temperature reductions followed by rain, hail, or both. By July, chicks are larger in size and are more independent of the brood hen [78]. If chicks are too large to be effectively brooded, severe monsoonal storms may result in chicks becoming soaked by rain and/or losing body temperature due to low temperatures or hail. While the Idaho study area is not in a monsoonal zone, it is possible that occasional severe July storms could produce similar effects. However, by July chicks should be more capable of thermoregulation relative to the early development period in June. Thus, we conclude that if exposure were a major source of chick mortality, models including the effect of June precipitation or the minimum temperature in June would have performed better.

Alternatively, JP may negatively affect sage-grouse chick survival through an interaction between increased moisture/humidity and predator search efficiency (i.e., moisture facilitated predation hypothesis). Moisture on skin and feathers increases bacterial activity, subsequently increasing scent production [36], [37], [38], [39], [40]. Mammalian predators have been shown to respond rapidly to the presence of prey odor [79] and increased scent production may lead to enhanced prey detection rates. A number of studies have found increased nest predation rates following precipitation events for greater sage-grouse and other gallinaceous birds [33], [80], [81], and the phenomenon of moisture-facilitated predation may apply to chicks and adult birds as well [34], [82]. We do not present observed chick predation rates due to concerns about correctly distinguishing between predation and scavenging. However, both of our study sites were inhabited by a suite of potential mammalian predators. Both study sites received predator management to reduce coyote (*Canis latrans*) predation on livestock, but coyotes and other common predators of sage-grouse chicks (red fox, badger [*Taxidea taxus*], weasels [*Mustella* sp.], and rattlesnakes [*Crotalus viridis*]) were present on both sites. In addition to the potential effects of moisture-facilitated predation by olfactory predators, JP may increase predation by avian predators if sage-grouse broods move to areas with less sagebrush cover following precipitation events to expedite drying and/or warming. Although not formally documented, we did observe broods along roadways at a higher frequency following precipitation events than at other times.

Effects of climate change on precipitation are less clear than the effects on temperature [72]. Climate models are inconclusive as to the sign of the effect on precipitation [83], and effects may vary by season [84]. In the absence of a consensus about effects of climate change on summer precipitation, anticipating the effect of changing precipitation on ecological communities and populations is difficult. Our analysis indicates that a 2 cm change in JP (positive or negative) would result in an approximate 15% change in sage-grouse chick survival (Fig. 4).

Sage-grouse are a species of great conservation concern in western North America. Chick survival has been shown to be an important determinant of population growth rates [9], [10], yet relatively little is known about climatic or other large-scale environmental factors affecting survival rates. Previous studies have identified specific habitat characteristics that influence survival [17], [18]. These studies have led to a proliferation of efforts to manage brood-rearing habitats without thorough consideration of the abiotic factors influencing both habitat quality and chick survival. Our study clearly demonstrates that large-scale abiotic factors such as drought, temperature and precipitation have significant effects on chick survival. These factors are beyond the control of state and federal wildlife management agencies and highlight the importance of considering current and future climatic conditions when developing policy and conservation strategies for this species. However, the effects we observed were measured for populations inhabiting large intact tracts of sagebrush habitat. The availability of adequate amounts of suitable habitat is a prerequisite that must be met for the effects of the abiotic factors we studied to be relevant.

## Supporting Information

Table S1 **Models for effect of age on greater sage-grouse chick survival.**
(DOCX)

Table S2 **Models for effect of hen age and hatch date on greater sage-grouse chick survival.** ‘Age’ is the top age varying model from Table S1. Signs in parentheses indicate the direction of respective covariate effects excluding chick age.
(DOCX)

Table S3 **Models for the effects of habitat greenness as measured by the Normalized Difference Vegetation Index (NDVI) on greater sage-grouse chick survival.** Signs in parentheses indicate the direction of respective covariate effects excluding chick age. All models (except the intercept only model) contain the base effects of quadratic chick age and hen age. Models were evaluated using the Quasi-Akaike's Information Criterion (QAIC). K = number of parameters. w_i_ = model weight (i.e. the likelihood of a particular model being the best model). R-score = percent reduction of deviance relative to the base model (Quadratic Chick Age+Hen Age).
(DOCX)

Table S4 **Parameter estimates with 95% confidence intervals for the top model of the effects of Normalized Difference Vegetation Index (NDVI) on greater sage-grouse chick survival.** Confidence intervals were calculated based on 5,000 bootstraps of the original data set.
(DOCX)

Table S5 **Models for the effects of drought on greater sage-grouse chick survival.** Signs in parentheses indicate the direction of respective covariate effects excluding chick age. All models (except the intercept only model) contain the base effects of quadratic chick age and hen age. Models were evaluated using the Quasi-Akaike's Information Criterion (QAIC). K = number of parameters. w_i_ = model weight (i.e. the likelihood of a particular model being the best model). R-score = percent reduction of deviance relative to the base model (Quadratic Chick Age+Hen Age).
(DOCX)

Table S6 **Parameter estimates with 95% confidence intervals for the top model of the effect of drought on greater sage-grouse chick survival.** Confidence intervals were calculated based on 5,000 bootstraps of the original data set.
(DOCX)

Table S7 **Models for the effects of climate on greater sage-grouse chick survival.** Signs in parentheses indicate the direction of respective covariate effects excluding chick age. All models (except the intercept only model) contain the base effects of quadratic chick age and hen age. Models were evaluated using the Quasi-Akaike's Information Criterion (QAIC). K = number of parameters. w_i_ = model weight (i.e. the likelihood of a particular model being the best model). R-score = percent reduction of deviance relative to the base model (Quadratic Chick Age+Hen Age).
(DOCX)

Table S8 **Parameter estimates with 95% confidence intervals for the top model of the effects of climate on greater sage-grouse chick survival.** Confidence intervals were calculated based on 5,000 bootstraps of the original data set.
(DOCX)

Table S9 **Evaluation of the effects of site and year effects on the top model from Table S7.** All models contain the base effects of quadratic chick age and hen age. Models were evaluated using the Quasi-Akaike's Information Criterion (QAIC). K = number of parameters. w_i_ = model weight (i.e. the likelihood of a particular model being the best model).
(DOCX)
