# Supplementary material for: Effects of Landscape-Scale Environmental Variation on Greater Sage-Grouse Chick Survival
Source: PLoS One. 2013 Jun 18;8(6):e65582. doi: 10.1371/journal.pone.0065582 (PMC3688806; doi:10.1371/journal.pone.0065582)
Supplement: Table S1 — Models for effect of age on greater sage-grouse chick survival. (DOCX) [file pone.0065582.s001.docx]

**Table S1.** Models for effect of age on greater sage-grouse chick survival.

| Model | K | QAICc | ΔQAICc | w_i_ |
| --- | --- | --- | --- | --- |
| Quadratic Chick Age Effect | 4 | 259.57 | 0.00 | 0.999 |
| Linear Chick Age Effect | 3 | 288.97 | 29.40 | 0.000 |
| Days 1-15 + Days 16-42 | 3 | 291.39 | 31.83 | 0.000 |
| Days 1-10 + Days 11-42 | 3 | 403.82 | 144.25 | 0.000 |
| Days 1-20 + Days 21-42 | 3 | 410.04 | 150.47 | 0.000 |
| Days 1-15 + Days 16-30 + Days 31-42 | 4 | 514.91 | 255.35 | 0.000 |
| Intercept only | 2 | 810.31 | 550.75 | 0.000 |
