# Supplementary material for: Effects of Landscape-Scale Environmental Variation on Greater Sage-Grouse Chick Survival
Source: PLoS One. 2013 Jun 18;8(6):e65582. doi: 10.1371/journal.pone.0065582 (PMC3688806; doi:10.1371/journal.pone.0065582)
Supplement: Table S2 — Models for effect of hen age and hatch date on greater sage-grouse chick survival. ‘Age’ is the top age varying model from Table S1. Signs in parentheses indicate the direction of respective covariate effects excluding chick age. (DOCX) [file pone.0065582.s002.docx]

**Table S2.** Models for effect of hen age and hatch date on greater sage-grouse chick survival. 'Age' is the top age varying model from Table A1. Signs in parentheses indicate the direction of respective covariate effects excluding chick age.

| Model | K | QAICc | ΔQAICc | w_i_ |
| --- | --- | --- | --- | --- |
| Chick Age + Hen Age (-) | 5 | 183.48 | 0.00 | 0.666 |
| Chick Age + Hen Age + Hatch Date (-,-) | 6 | 184.87 | 1.39 | 0.333 |
| Chick Age | 4 | 259.57 | 76.09 | 0.000 |
| Chick Age + Hatch Date (-) | 5 | 260.91 | 77.43 | 0.000 |
| Intercept only | 2 | 810.31 | 626.83 | 0.000 |
