# Supplementary material for: Effects of Landscape-Scale Environmental Variation on Greater Sage-Grouse Chick Survival
Source: PLoS One. 2013 Jun 18;8(6):e65582. doi: 10.1371/journal.pone.0065582 (PMC3688806; doi:10.1371/journal.pone.0065582)
Supplement: Table S3 — Models for the effects of habitat greenness as measured by the Normalized Difference Vegetation Index (NDVI) on greater sage-grouse chick survival. Signs in parentheses indicate the direction of respective covariate effects excluding chick age. All models (except the intercept only model) contain the base effects of quadratic chick age and hen age. Models were evaluated using the Quasi-Akaike's Information Criterion (QAIC). K = number of parameters. wi = model weight (i.e. the likelihood of a particular model being the best model). R-score = percent reduction of deviance relative to the base model (Quadratic Chick Age+Hen Age). (DOCX) [file pone.0065582.s003.docx]

**Table S3.** Models for the effects of habitat greenness as measured by the Normalized Difference Vegetation Index (NDVI) on greater sage-grouse chick survival. Signs in parentheses indicate the direction of respective covariate effects excluding chick age. All models (except the intercept only model) contain the base effects of quadratic chick age and hen age. Models were evaluated using the Quasi-Akaike’s Information Criterion (QAIC). K = number of parameters. w_i_ = model weight (i.e. the likelihood of a particular model being the best model). R-score = percent reduction of deviance relative to the base model (Quadratic Chick Age + Hen Age).

| Model | K | QAICc | ΔQAICc | w_i_ | R-score |
| --- | --- | --- | --- | --- | --- |
| July Mean NDVI (+) | 6 | 178.53 | 0.00 | 0.221 | 0.022 |
| Summer Maximum NDVI (+) | 6 | 179.33 | 0.79 | 0.149 | 0.019 |
| July Maximum NDVI (+) | 6 | 179.46 | 0.93 | 0.139 | 0.019 |
| June Maximum NDVI (+) | 6 | 180.32 | 1.78 | 0.091 | 0.016 |
| May Mean NDVI (+) | 6 | 180.39 | 1.86 | 0.087 | 0.016 |
| NDVI on Day of Hatch (+) | 6 | 180.80 | 2.27 | 0.071 | 0.015 |
| NDVI 15 Days Post-Hatch (+) | 6 | 181.04 | 2.50 | 0.063 | 0.014 |
| Summer Mean NDVI (+) | 6 | 181.21 | 2.68 | 0.058 | 0.013 |
| June Mean NDVI (+) | 6 | 182.83 | 4.30 | 0.026 | 0.008 |
| NDVI on Day of Observation (+) | 6 | 183.05 | 4.52 | 0.023 | 0.008 |
| NDVI 10 Days Prior to Observation (+) | 6 | 183.19 | 4.66 | 0.022 | 0.007 |
| NDVI 5 Days Prior to Observation (+) | 6 | 183.19 | 4.66 | 0.022 | 0.007 |
| Chick Age + Hen Age (-) | 5 | 183.48 | 4.95 | 0.019 | 0.000 |
| May Maximum NDVI (+) | 6 | 184.76 | 6.23 | 0.010 | 0.002 |
| Intercept only | 2 | 810.31 | 631.78 | 0.000 | ------- |
