# Supplementary material for: Effects of Landscape-Scale Environmental Variation on Greater Sage-Grouse Chick Survival
Source: PLoS One. 2013 Jun 18;8(6):e65582. doi: 10.1371/journal.pone.0065582 (PMC3688806; doi:10.1371/journal.pone.0065582)
Supplement: Table S4 — Parameter estimates with 95% confidence intervals for the top model of the effects of Normalized Difference Vegetation Index (NDVI) on greater sage-grouse chick survival. Confidence intervals were calculated based on 5,000 bootstraps of the original data set. (DOCX) [file pone.0065582.s004.docx]

**Table S4.** Parameter estimates with 95% confidence intervals for the top model of the effects of Normalized Difference Vegetation Index (NDVI) on greater sage-grouse chick survival. Confidence intervals were calculated based on 5,000 bootstraps of the original data set.

| Parameter | Estimate | LCL | UCL |
| --- | --- | --- | --- |
| *d* | 1.6149 | 1.3731 | 2.3456 |
| Intercept | 3.4968 | 2.6405 | 4.2546 |
| Linear Chick Age | 0.1087 | -0.0065 | 0.1598 |
| Quadratic Chick Age | -0.0013 | -0.0027 | 0.0016 |
| Hen Age | -0.6807 | -1.2838 | 0.0339 |
| July Average NDVI | 0.0681 | -0.2343 | 0.2340 |
