# Supplementary material for: Effects of Landscape-Scale Environmental Variation on Greater Sage-Grouse Chick Survival
Source: PLoS One. 2013 Jun 18;8(6):e65582. doi: 10.1371/journal.pone.0065582 (PMC3688806; doi:10.1371/journal.pone.0065582)
Supplement: Table S5 — Models for the effects of drought on greater sage-grouse chick survival. Signs in parentheses indicate the direction of respective covariate effects excluding chick age. All models (except the intercept only model) contain the base effects of quadratic chick age and hen age. Models were evaluated using the Quasi-Akaike's Information Criterion (QAIC). K = number of parameters. wi = model weight (i.e. the likelihood of a particular model being the best model). R-score = percent reduction of deviance relative to the base model (Quadratic Chick Age+Hen Age). (DOCX) [file pone.0065582.s005.docx]

**Table S5.** Models for the effects of drought on greater sage-grouse chick survival. Signs in parentheses indicate the direction of respective covariate effects excluding chick age. All models (except the intercept only model) contain the base effects of quadratic chick age and hen age. Models were evaluated using the Quasi-Akaike’s Information Criterion (QAIC). K = number of parameters. w_i_ = model weight (i.e. the likelihood of a particular model being the best model). R-score = percent reduction of deviance relative to the base model (Quadratic Chick Age + Hen Age).

| Model | K | QAICc | ΔQAICc | w_i_ | R-score |
| --- | --- | --- | --- | --- | --- |
| Winter PZI (+) | 6 | 58.21 | 0.00 | 0.961 | 0.396 |
| May PZI (+) | 6 | 64.64 | 6.43 | 0.038 | 0.376 |
| June PZI (+) | 6 | 72.67 | 14.46 | 0.000 | 0.351 |
| Summer PZI (+) | 6 | 110.15 | 51.94 | 0.000 | 0.235 |
| June PDSI (+) | 6 | 127.23 | 69.03 | 0.000 | 0.181 |
| July PDSI (+) | 6 | 130.47 | 72.26 | 0.000 | 0.171 |
| Summer PDSI (+) | 6 | 133.85 | 75.64 | 0.000 | 0.161 |
| May PDSI (+) | 6 | 141.65 | 83.44 | 0.000 | 0.137 |
| July PZI (+) | 6 | 154.46 | 96.25 | 0.000 | 0.097 |
| Winter PDSI (+) | 6 | 176.51 | 118.30 | 0.000 | 0.028 |
| Chick Age + Hen Age (-) | 5 | 183.48 | 125.27 | 0.000 | 0.000 |
| Intercept only | 2 | 810.31 | 752.10 | 0.000 | ------- |
