# Supplementary material for: Effects of Landscape-Scale Environmental Variation on Greater Sage-Grouse Chick Survival
Source: PLoS One. 2013 Jun 18;8(6):e65582. doi: 10.1371/journal.pone.0065582 (PMC3688806; doi:10.1371/journal.pone.0065582)
Supplement: Table S7 — Models for the effects of climate on greater sage-grouse chick survival. Signs in parentheses indicate the direction of respective covariate effects excluding chick age. All models (except the intercept only model) contain the base effects of quadratic chick age and hen age. Models were evaluated using the Quasi-Akaike's Information Criterion (QAIC). K = number of parameters. wi = model weight (i.e. the likelihood of a particular model being the best model). R-score = percent reduction of deviance relative to the base model (Quadratic Chick Age+Hen Age). (DOCX) [file pone.0065582.s007.docx]

**Table S7.** Models for the effects of climate on greater sage-grouse chick survival. Signs in parentheses indicate the direction of respective covariate effects excluding chick age. All models (except the intercept only model) contain the base effects of quadratic chick age and hen age. Models were evaluated using the Quasi-Akaike’s Information Criterion (QAIC). K = number of parameters. w_i_ = model weight (i.e. the likelihood of a particular model being the best model). R-score = percent reduction of deviance relative to the base model (Quadratic Chick Age + Hen Age).

| Model | K | QAICc | ΔQAICc | w_i_ | R-score |
| --- | --- | --- | --- | --- | --- |
| May Min Temp + July Precip (-,-) | 7 | -58.30 | 0.00 | 0.999 | 0.766 |
| May Max Temp + June Min Temp (-,+) | 7 | -14.01 | 44.29 | 0.000 | 0.628 |
| May Max Temp + June Max Temp (-,-) | 7 | -12.65 | 45.65 | 0.000 | 0.623 |
| May Max Temp (-) | 6 | -10.46 | 47.84 | 0.000 | 0.610 |
| July Precip (-) | 6 | -9.46 | 48.84 | 0.000 | 0.607 |
| May Min Temp + Winter Precip (-,+) | 7 | 8.75 | 67.05 | 0.000 | 0.557 |
| Winter Precip (+) | 6 | 47.95 | 106.26 | 0.000 | 0.428 |
| May Min Temp + July Max Temp (-,+) | 7 | 52.42 | 110.72 | 0.000 | 0.421 |
| June Min Temp + June Precip (+,+) | 7 | 75.17 | 133.47 | 0.000 | 0.350 |
| May Min Temp + June Min Temp (-,+) | 7 | 89.37 | 147.67 | 0.000 | 0.306 |
| June Precip (+) | 6 | 89.70 | 148.00 | 0.000 | 0.298 |
| May Min Temp (-) | 6 | 91.41 | 149.71 | 0.000 | 0.293 |
| May Precip + July Max Temp (+,+) | 7 | 108.55 | 166.85 | 0.000 | 0.246 |
| May Precip (+) | 6 | 118.64 | 176.94 | 0.000 | 0.208 |
| June Max Temp + July Max Temp (-,+) | 7 | 119.74 | 178.04 | 0.000 | 0.211 |
| July Max Temp (+) | 6 | 156.93 | 215.23 | 0.000 | 0.089 |
| June Max Temp (-) | 6 | 167.73 | 226.03 | 0.000 | 0.055 |
| June Min Temp (+) | 6 | 179.44 | 237.74 | 0.000 | 0.019 |
| Chick Age + Hen Age (-) | 5 | 183.48 | 241.78 | 0.000 | 0.000 |
| Intercept only | 2 | 810.31 | 868.61 | 0.000 | ------- |
