# Supplementary material for: Effects of Landscape-Scale Environmental Variation on Greater Sage-Grouse Chick Survival
Source: PLoS One. 2013 Jun 18;8(6):e65582. doi: 10.1371/journal.pone.0065582 (PMC3688806; doi:10.1371/journal.pone.0065582)
Supplement: Table S8 — Parameter estimates with 95% confidence intervals for the top model of the effects of climate on greater sage-grouse chick survival. Confidence intervals were calculated based on 5,000 bootstraps of the original data set. (DOCX) [file pone.0065582.s008.docx]

**Table S8.** Parameter estimates with 95% confidence intervals for the top model of the effects of climate on greater sage-grouse chick survival. Confidence intervals were calculated based on 5,000 bootstraps of the original data set.

| Parameter | Estimate | LCL | UCL |
| --- | --- | --- | --- |
| *d* | 1.7085 | 1.3850 | 2.4379 |
| Intercept | 3.1456 | 2.1258 | 4.0126 |
| Linear Chick Age | 0.1298 | 0.0069 | 0.1733 |
| Quadratic Chick Age | -0.0014 | -0.0028 | 0.0013 |
| Hen Age | -0.4696 | -1.1274 | 0.3227 |
| May Minimum Temperature | -0.2125 | -0.4481 | 0.1176 |
| July Precipitation | -0.3664 | -0.6830 | -0.1207 |
