# Supplementary material for: Effects of Landscape-Scale Environmental Variation on Greater Sage-Grouse Chick Survival
Source: PLoS One. 2013 Jun 18;8(6):e65582. doi: 10.1371/journal.pone.0065582 (PMC3688806; doi:10.1371/journal.pone.0065582)
Supplement: Table S9 — Evaluation of the effects of site and year effects on the top model from Table S7. All models contain the base effects of quadratic chick age and hen age. Models were evaluated using the Quasi-Akaike's Information Criterion (QAIC). K = number of parameters. wi = model weight (i.e. the likelihood of a particular model being the best model). (DOCX) [file pone.0065582.s009.docx]

**Table S9.** Evaluation of the effects of site and year effects on the top model from Table A7. All models contain the base effects of quadratic chick age and hen age. Models were evaluated using the Quasi-Akaike’s Information Criterion (QAIC). K = number of parameters. w_i_ = model weight (i.e. the likelihood of a particular model being the best model).

| Model | K | QAICc | ΔQAICc | w_i_ |
| --- | --- | --- | --- | --- |
| May Min Temp + July Precip | 7 | -58.30 | 0.00 | 0.538 |
| May Min Temp + July Precip + Site | 8 | -57.37 | 0.93 | 0.337 |
| MMT + JP + Site + MMT*Site + JP*Site | 10 | -55.39 | 2.91 | 0.125 |
| May Min Temp + July Precip + Year | 15 | DID NOT CONVERGE | | |
